# Eyes of Things

**DOI:** 10.3390/s17051173

**Published:** 2017-05-21

**Authors:** Oscar Deniz, Noelia Vallez, Jose L. Espinosa-Aranda, Jose M. Rico-Saavedra, Javier Parra-Patino, Gloria Bueno, David Moloney, Alireza Dehghani, Aubrey Dunne, Alain Pagani, Stephan Krauss, Ruben Reiser, Martin Waeny, Matteo Sorci, Tim Llewellynn, Christian Fedorczak, Thierry Larmoire, Marco Herbst, Andre Seirafi, Kasra Seirafi

**Affiliations:** 1 VISILAB, University of Castilla-La Mancha, E.T.S.I.Industriales, Avda Camilo Jose Cela s/n, Ciudad Real 13071, Spain; noelia.vallez@uclm.es (N.V.); JoseL.espinosa@uclm.es (J.L.E.-A.); JoseMaria.Rico@uclm.es (J.M.R.-S.); Javier.Parra@uclm.es (J.P.-P.); gloria.bueno@uclm.es (G.B.); 2 Movidius, 1st Floor, O’Connell Bridge House, D’Olier Street, Dublin 2, Ireland; david.moloney@movidius.com (D.M.); alireza.dehghani@movidius.com (A.D.); aubrey.dunne@movidius.com (A.D.); 3 DFKI, Augmented Vision Research Group, Tripstaddterstr. 122, 67663 Kaiserslautern, Germany; alain.pagani@dfki.de (A.P.); stephan.krauss@dfki.de (S.K.); ruben.reiser@dfki.de (R.R.); 4 Awaiba, Madeira Tecnopolo, 9020-105 Funchal, Portugal; waeny@awaiba.com; 5 nViso SA, PSE-D, Site EPFL, CH-1015 Lausanne, Switzerland; matteo.sorci@nviso.ch (M.S.); tim.llewellynn@nviso.ch (T.L.); 6 THALES Communications & Security, 4 Avenue des Louvresses, 92230 Gennevilliers, France; christian.fedorczak@thalesgroup.com (C.F.); thierry.larmoire@thalesgroup.com (T.L.); 7 Evercam, 6-7 Granby Row, Dublin 1, D01 FW20, Ireland; marco@evercam.io; 8 Fluxguide, Burggasse 7-9/9, 1070 Vienna, Austria; andre@fluxguide.com (A.S.); kasra@fluxguide.com (K.S.)

**Keywords:** embedded computer vision, eyes of things, Internet of Things, computer vision

## Abstract

Embedded systems control and monitor a great deal of our reality. While some “classic” features are intrinsically necessary, such as low power consumption, rugged operating ranges, fast response and low cost, these systems have evolved in the last few years to emphasize connectivity functions, thus contributing to the Internet of Things paradigm. A myriad of sensing/computing devices are being attached to everyday objects, each able to send and receive data and to act as a unique node in the Internet. Apart from the obvious necessity to process at least some data at the edge (to increase security and reduce power consumption and latency), a major breakthrough will arguably come when such devices are endowed with some level of autonomous “intelligence”. Intelligent computing aims to solve problems for which no efficient exact algorithm can exist or for which we cannot conceive an exact algorithm. Central to such intelligence is Computer Vision (CV), i.e., extracting meaning from images and video. While not everything needs CV, visual information is the richest source of information about the real world: people, places and things. The possibilities of embedded CV are endless if we consider new applications and technologies, such as deep learning, drones, home robotics, intelligent surveillance, intelligent toys, wearable cameras, etc. This paper describes the Eyes of Things (EoT) platform, a versatile computer vision platform tackling those challenges and opportunities.

## 1. Introduction

Sensors are central to the Internet of Things paradigm. A special place is reserved for imaging sensors, which represent one of the richest sources of information about the world. In this respect, computer vision (i.e., the automatic processing and analysis of data captured by imaging sensors) is a field that was traditionally focused on factory automation, although it is now rapidly moving beyond academic research and factories to many novel application scenarios. However, while computer vision is a mature research field from a theoretical point of view, practical ubiquitous vision has not progressed comparably. The particular case of vision in the Internet of Things paradigm represents a fundamental challenge. Image analysis and inference typically require significant computing power. However, the sheer volume of visual information that can be potentially generated by mobile devices cannot be transferred to the cloud for processing. This problem gets even worse when we consider emerging sensing technologies like 3D and hyperspectral cameras. When we consider embedded devices, wirelessly transmitting data for remote computation can cost orders of magnitude more energy per operation compared to processing locally in a device. Processing locally, on the other hand, there is the risk of drawing such amounts of energy that practical mobile/wearable applications are precluded.

All of this, coupled with an increasing awareness about the risks of uploading sensitive data under most interpretations, the image of an identifiable person is considered personal data and as such is subject to legal regulation and protection calls for efforts at edge processing [1].

In general, in the last few years, cognitive applications and services have been acknowledged as key drivers of innovation and demand. Clearly, the new intelligent features provided will determine competitiveness. This is already being widely recognized, and demand is growing worldwide for Artificial Intelligence (AI) experts and technologies. Estimates point to an impressive AI market growth of nearly 63% to 2022 [2]. AI is poised to become a transformative technology with an unprecedented impact on our society. In this context, computer vision (in itself one of the most respected branches of artificial intelligence) should play a major role.

This work describes Eyes of Things (EoT) [3], a novel reference platform designed from the ground up for flexible mobile computer vision. The platform can be used independently and also embedded into all types of artefacts. Carefully selected hardware and software technologies allow maximizing inferred information per milliwatt. Coupled with low-power connectivity hardware and lightweight protocols, it will allow creating innovative applications and services that go beyond what current vision systems can do. The platform is targeted at OEMs (Original Equipment Manufacturers). Flexibility comes in a number of ways. Open source code and specifications allow for wide usage and modifications tailored to the needs of each application or service. Flexibility also reflects in the ease of use of the device, which allows for close interaction with everyday objects such as smartphones and with cloud services.

This paper is organized as follows. Section 2 reviews previous related work. Section 3 and Section 4 describe the hardware and software of EoT. Section 5 describes the results obtained, as well as a number of demonstrators built with the platform. Finally, Section 6 outlines the conclusions of this work.

## 2. State of the Art

Historically, work in wireless and distributed camera networks lies conceptually close to the outlined Internet of Things scenario that envisages tiny connected cameras everywhere. Vision sensor networks aim at small power-efficient vision sensor nodes that can operate as standalone. This is a natural evolution of the significant research effort made in the last few years on Wireless Sensor Networks (WSN).

Research on Vision WSNs (VWSN) has been significant (see surveys [4,5,6,7,8,9,10]). Platforms can be roughly divided into two groups:

- Computer vision platform, i.e., no video streaming.
- Streaming platform, with computer vision used to improve compression or stream only on interesting events; these have been also called ‘multimedia wireless sensor networks’.

The first category is driven by the computer vision side, while the second category is more related to efficient communications. Within the first category, the three basic required capabilities for a node are: capture, process and transmit. Another possible classification is based on application. Thus, three basic levels of operation have been proposed in this context: Tier-1: object/person detection; Tier-2: object/person recognition; and Tier-3: object/person tracking. These levels can operate in a hierarchical manner. Tier-1 applications can be achieved with low cost and low energy dissipation platforms. Tier-3 may involve streaming video, which requires comparably more energy.

Early sensor networks were mostly confined to research environments (wireless smart camera networks for the surveillance of public spaces). Platforms are typically demonstrated in a specific application (surveillance, tracking, etc.). They are generally assessed in terms of energy consumption and the complexity of the demonstrated computer vision task. While these aspects are obviously important, a development platform requires attention to other aspects such as operating system, programming, vision libraries and interoperability with existing networks. As a consequence, existing VWSN platforms are mostly research-level prototypes that use non-mainstream network technologies (6LoWPAN, ZigBee, IEEE 802.15.4, etc.) and exotic operating systems, languages and tool chains (TinyOS, Contiki, nesC, etc.). This has prevented widespread adoption and commercialization, which has been recently observed by some researchers [11]: “Given the fair number of proposed designs, it is somewhat surprising that a general-purpose embedded smart camera, based on an open architecture is difficult to find, and even more difficult to buy.”

In [12], the authors already considered the potential of wireless and distributed camera networks in the emerging IoT paradigm. In that work, it was clearly concluded that small form-factor low-power cameras can actually open up a wide range of new applications, and co-existence with cloud services is crucial. The work [13] also contended that ideas for new applications and services can thrive with flexible ‘mobile vision’, arguing that one of the best platforms available for this are camera-equipped consumer devices (i.e., smartphones and tablets). These devices have the computational resources to support computer vision algorithms, along with sensors such as compasses, accelerometers and gyroscopes that can support applications, such as augmented reality and geolocalization. Last but not least, they offer an easy-to-use user interface, touch screens being the most important element.

When we consider the IoT, for these vision devices to be everywhere, they have to be easy to program and easy to use. This means that, to converge with (and take advantage of) the Internet of Things paradigm, the node should use standardized and widespread Internet technologies and protocols (TCP/IP, WiFi, etc.) as much as possible, and much as most IoT devices can be easily accessed by anyone from everywhere (i.e., with smartphones), the node should be able to interact with these de facto user interfaces and also with increasingly powerful cloud-based analysis [14]. Likewise, low cost and programmability are required for widespread usage both at the developer and end-user levels. Thus, in this current scenario, it is not uncommon that researchers and engineers resort to low-end (if versatile, easy-to-use and low-cost) platforms. Some recent examples of vision projects using smartphones are described in [15,16,17]. In [18], the authors proposed a Raspberry Pi+camera system for a wireless smart camera mesh network. The Raspberry Pi has been also successfully used commercially in the Placemeter (now part of NETGEAR) ‘urban intelligence’ platform www.placemeter.com.

EoT can be seen as a low-power, low-cost small camera module. Still, it aims at going beyond that by aligning itself with the IoT paradigm. On the one hand, it integrates nicely with massively-used user interfaces in the form of smartphones and tablets. On the other hand, it also integrates with additional capabilities that can be only obtained in the cloud. Finally, it makes a concerted effort to keep power consumption low, not only at the hardware level, but also considering software and communication protocols. In practice, achieving this has required an important development effort on multiple fronts, trading-off some features for others. Clearly, optimized computing performance can be achieved at the cost of very low programmability and difficult interaction with both developers and end-users (the recent PULP vision system [19], for example, can run a carefully optimized ConvNet-based detector for smart surveillance on a few mJ). On the other hand, programmability and low cost are clearly ‘optimized’ in other platforms, such as the Raspberry Pi [20].

A few flexible embedded vision platforms have been proposed recently such as JeVOIS [21] and OpenMV [22], both successful Kickstarter campaigns. Both platforms aim at a low-cost extensible and easy-to-use embedded vision platform. However, these approaches fall short of the demanding requirements for innovative applications that include power-hungry methodologies, such as the deep learning paradigm. The JeVOIS platform, for example, draws 3.5 Watts of power, and in fact, a significantly large portion of the device volume is occupied by a large cooling fan. JeVOIS does not include wireless connectivity, while a separate WiFi shield is available in the case of OpenMV. On the other hand, NVIDIA™ platforms have a strong software suite and are much more capable, supporting deep learning networks. However, systems based on these platforms often cost thousands of dollars, which is clearly beyond the reach of most users.

From the industry side, there have been cases of successful vision-based consumer products, the best example being Microsoft’s Kinect, which combined sensing hardware with computer vision techniques to create a video gaming device [23]. Kinect still stands as the fastest selling consumer electronics device. Other similar examples are Dyson’s vacuum cleaners, Google Glass, Microsoft HoloLens, Amazon Fire, etc. Nevertheless, these and other examples have generally involved large companies that can afford the required specific designs. Arguably, no flexible and affordable open platform for mobile and connected vision is currently available.

## 3. EoT Hardware

**Figure 1:**
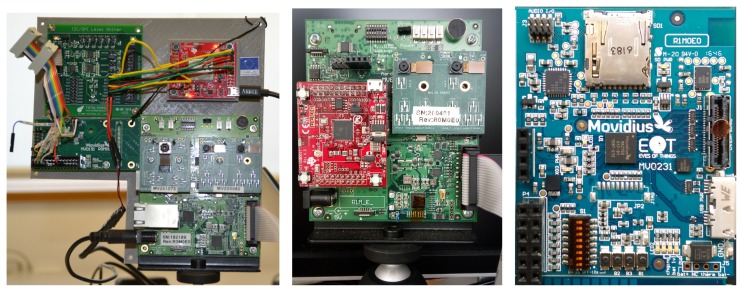
Development of Ears of Things (EoT) boards. Sizes, from left to right (in mm): 200 × 180, 100 × 100, 57 × 46.

**Figure 2:**
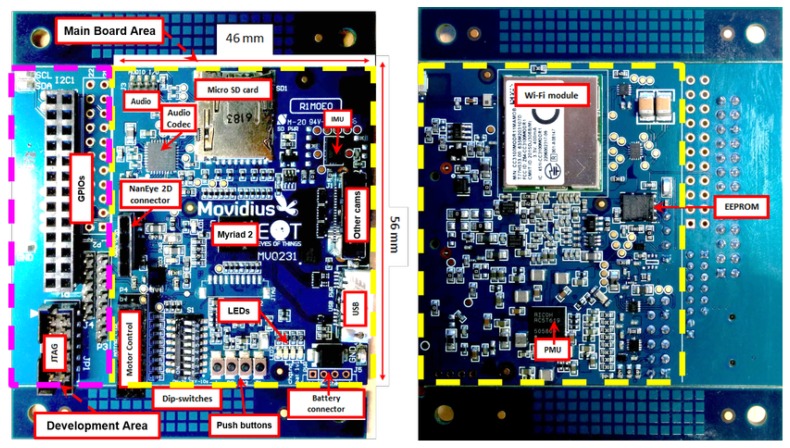
Top and rear views of the EoT board, showing the main components.

**Figure 3:**
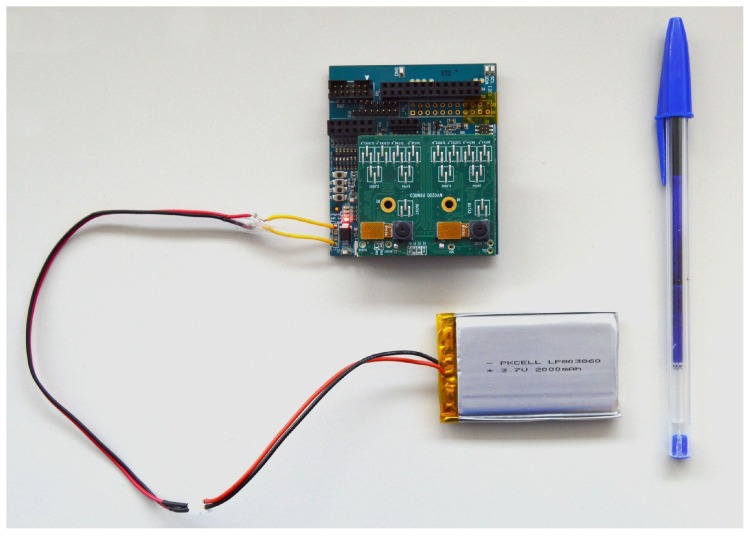
Board connected to a LiPo battery.

The EoT hardware has been developed in steps. Figure 1 shows the three iterations of the board. The first prototype developed (on the left) was based on a Movidius Myriad 2 development board. The MV0182 development board includes a Myriad 2 SoC plus other components, such as EEPROM, HDMI transmitter, Ethernet, SD card, IMU, IR and pressure sensors, etc. The board size is 90 mm × 90 mm, plus a number of additional interconnected boards. The successive board iterations either removed unused components or integrated external components into a single board. The board on the right is the factor-form version, which is a 57 mm × 46 mm eight-layer high-density PCB (Printed Circuit Board), optimized for low cost, size and power efficiency (see Figure 2 and Figure 3).

**Figure 4:**
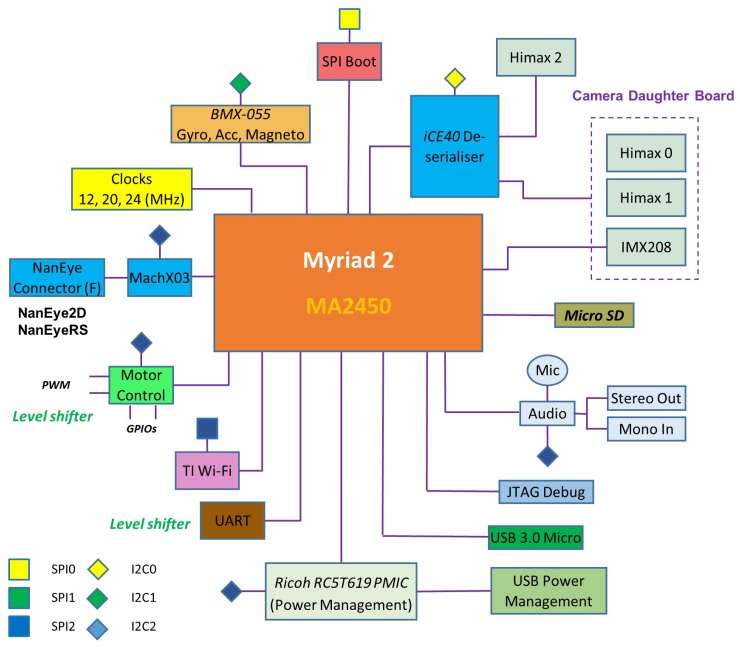
EoT block diagram.

**Figure 5:**
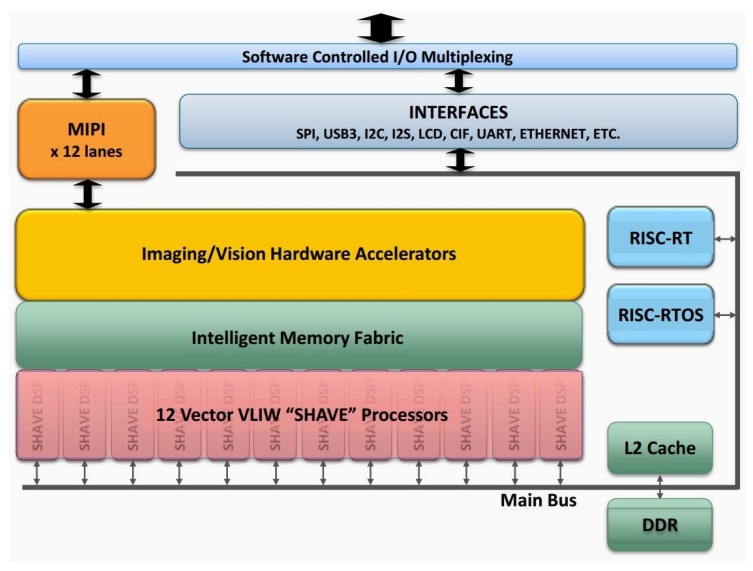
Myriad 2 SoC architecture.

A block diagram of the complete system is shown in Figure 4. All processing and control is performed by the low-power Myriad 2 MA2450 SoC by Movidius (an Intel company, Santa Clara, CA, USA); see [24]. This SoC has been designed from the ground up considering efficient mobile high-performance computation. Myriad 2 is a heterogeneous, multicore always-on SoC supporting computational imaging and visual awareness for mobile, wearable and embedded applications. Myriad 2 is based on the proprietary twelve 128-bit very long instruction word “SHAVE” processors, two 32-bit RISC processors (LeonOS and LeonRT) and a hardware acceleration pipeline backed by a shared multicore memory subsystem and peripherals (Figure 5). The chip includes 2 Mbytes of on-chip memory and 400 Gbytes per second of sustained internal memory bandwidth. It supports up to six full HD 60 frames per second camera inputs simultaneously via Mobile Industry Processor Interface (MIPI) lanes. It has been designed to operate at 0.9 V for nominal 600-MHz operation, and contains 20 different power-islands coupled with extensive clock-gating under a software API to minimize power dissipation. The twelve integrated SHAVE processors combined with video and ISP hardware accelerators achieve 1000 GFLOPs (fp16 type) at 600 mW including peripherals and 512 MB LP DDR3 DRAM die stacked in the package.

The Myriad 2 VPU incorporates parallelism, ISA (Instruction Set Architecture) and microarchitectural features such as multi-ported register-files and native hardware support for sparse data-structures, video hardware accelerators, configurable multicore and multiport memory banks; thus, it provides exceptional and highly sustainable performance efficiency across a range of computational imaging and computer vision applications, including those with low latency requirements on the order of milliseconds.

**Figure 6:**
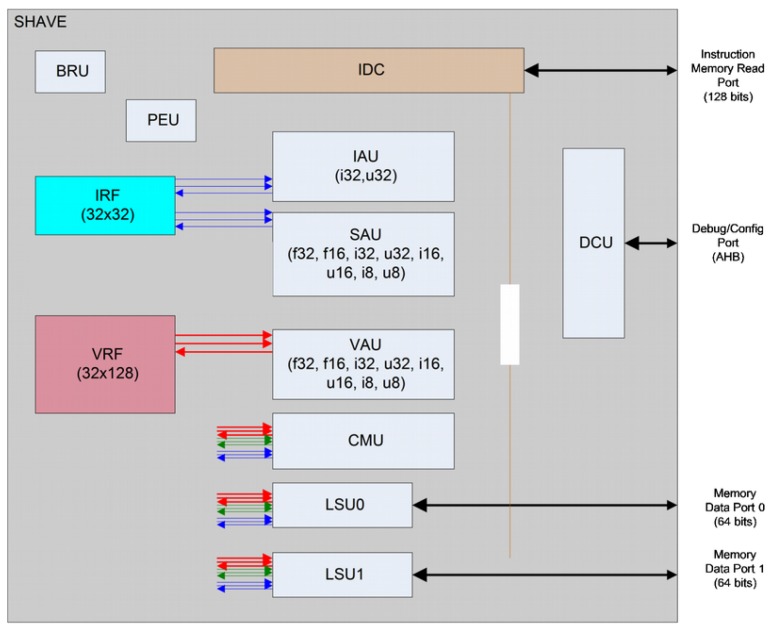
SHAVE internal architecture.

To guarantee sustained high performance and minimize power use, the aforementioned Movidius Streaming Hybrid Architecture Vector Engine (SHAVE) processor contains wide and deep register files coupled with a Very Long Instruction Word (VLIW) controlling multiple functional units including extensive SIMD capability for high parallelism and throughput at both the functional unit and processor level. The SHAVE processor is a hybrid stream processor architecture combining the best features of GPUs, DSPs and RISC with both 8-, 16- and 32-bit integer and 16- and 32-bit floating-point arithmetic, as well as unique features such as hardware support for sparse data structures. The architecture maximizes performance per watt while maintaining ease of programmability, especially in terms of support for design and porting of multicore software applications. Figure 6 shows the SHAVE internal architecture.

The functional units are: the Predicated Execution Unit (PEU), which is helpful for implementing conditional branching and also to make conditional stores on Load-Store Units LSU or Vector Arithmetic Units (VAU); the Branch and Repeat Unit (BRU) that provides functionality for branching; two 64-bit load-store units (LSU0 and LSU1), which provide functionality for loading and storing data to both register files; the 128-bit VAU that provides both floating point and integer operations on the VRF registers using 8-, 16- and 32-bit data types of both integer or floating point; the 32-bit Scalar Arithmetic Unit (SAU) provides floating point operations support on the IRF; the 32-bit Integer Arithmetic Unit (IAU) that provides integer operation support on the IRF registers, as well as support for different shifting and logic operations; and the 128-bit Compare Move Unit (CMU). Each of these units is enabled separately by a header in the variable length instruction.

**Figure 7:**
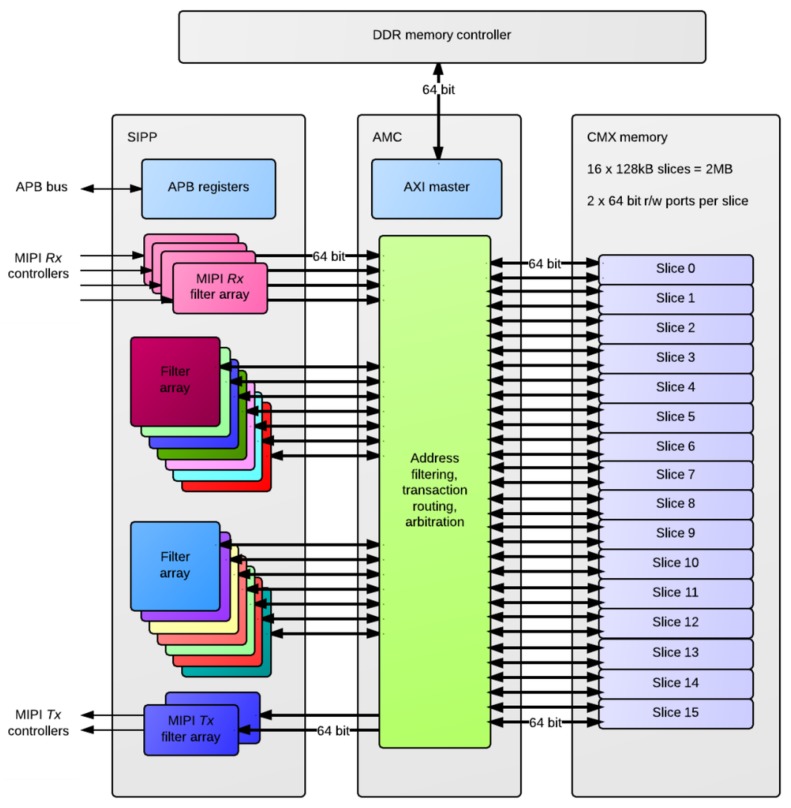
Block diagram of SIPP accelerator, AMC and connections to CMX.

A concerted effort was also made in Myriad2 to profile the key performance-limiting kernels and define SIPP hardware accelerators, as a component of Media Subsystem (MSS). The SIPP accelerators, see Figure 7, consist of a collection of hardware filters with a common programming interface, targeting Image Signal Processing (ISP) and Computer Vision (CV) applications over 1 TFLOPs of performance. These hardware accelerators support context switching in order to allow the processing of multiple parallel camera streams. The SIPP accelerators provide configurable hardware acceleration of computationally-intensive filter functions commonly used in ISP/CV applications, allowing this functionality to be offloaded from the SHAVEs. Figure 2 shows a high-level block diagram of the SIPP accelerators in the system context along with interfaces to MIPI Rx/Tx controllers, the APB slave interface, the AMC and CMX memory. The programmable SIPP hardware accelerators implemented in the Myriad 2 VPU include a poly-phase resizer, lens shading correction, Harris corner detector, histogram of oriented gradients edge operator, convolution filter, sharpening filter, gamma correction, tone mapping and luminance and chrominance denoising.

**Figure 8:**
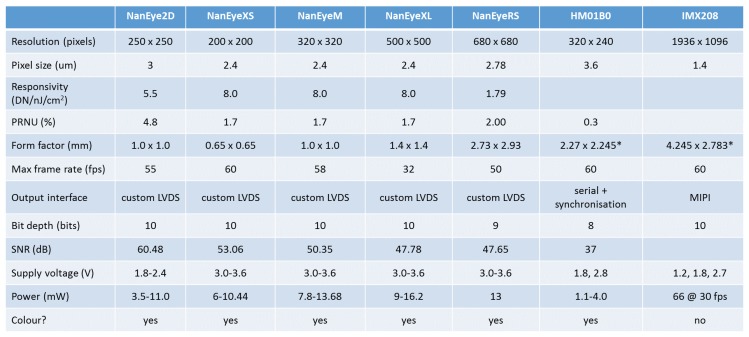
Cameras supported by EoT.

Figure 8 shows the cameras supported by EoT. The system supports always-on vision processing using a range of low-power visual sensors including AMS International AG/Awaiba NanEye2D, which is capable of capturing 250 × 250 pixel images and holds the record for the world’s most compact digital camera [25]. This is a module package CMOS image sensor and integrated lens that is fully self-timed, consuming less than 5 mW at 60 FPS. In addition, it supports NanEyeRS (680 × 680 pixel at 50 FPS 13 mW), Himax HM01B0 (320 × 320 pixel @30 FPS <2 mW; 160 × 120 pixel @30 FPS 1.1 mW) along with the Sony IMX208 high resolution sensor (1080p, 60 FPS) cameras. Cameras are connected to the board through a flex cable.

Additional peripherals such as a tri-axial gyroscope, tri-axial accelerometer, magnetometer and microphone enable a progressive activation approach for the processing of ‘interesting’ events. Based on the sensor data and the information extracted from visual processing and neural inference, decisions coupled with relevant metadata can be communicated via the integrated low-power WiFi module to external devices or to the cloud. The WiFi module is TI’s CC3100 [26], which is a low-power device designed for IoT applications. In personal assistant use-cases, audio cues for prompting and notification are enabled via a complete on-board audio codec. Integrated level shifters expose motor control pins for glue-less, yet generic, robotic control (GPIOs, PWMs, I2C, UART). Push buttons, LEDs, DIP-switches, USB and micro-SD functionality support rapid development and secure (see below) data logging modalities.

Power management is provided by a Ricoh RC5T619 PMIC. Power can be provided through a USB 3.0 type AB connector (5 V) or from a lithium ion battery (3.7 V). The device has an internal battery charger and battery status/charging indicator. More details about the EoT hardware can be found in [3].

## 4. EoT Software

Embedded systems are generally difficult to use. While the hardware provides the core functionality, the use of the device in innovative applications crucially depends on available software. Therefore, a software stack has been developed to complete the platform totalling, at the time of writing, 1.4 million lines of code.

**Figure 9:**
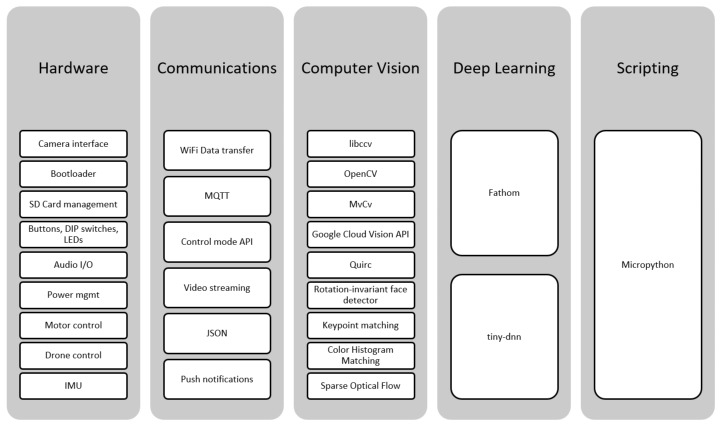
Main EoT software modules.

The software has been developed on top of RTEMS and the Movidius’ Development Kit (MDK). Figure 9 shows an overview of the main software modules. A number of modules allow interaction with the hardware components. WiFi-based communication functionality allows interaction with the outside world (thus providing the ‘of Things’ qualification). Computer vision and deep learning (more precisely, convolutional neural networks) provide the tools to create intelligent applications. Finally, scripting provides an easy way to develop prototypes.

In the following subsections, the major software modules are described in more detail.

### 4.1. Interaction with Hardware

#### 4.1.1. Bootloader

**Figure 10:**
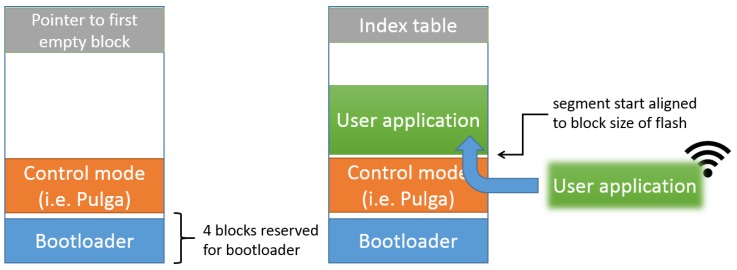
Flash memory layout.

The EoT bootloader allows either booting into the so-called “control mode” (see below) or loading and running a user application (which can be uploaded and stored in the device EEPROM memory when in control mode). The choice of what to boot is made through the use of a DIP switch on the device. The bootloader itself, the control mode software and the user apps are all stored in the device’s EEPROM (flash) memory. The bootloader uses a filesystem on the flash memory. Figure 10 shows the flash memory layout that is used. The bootloader is always stored at the beginning of the flash memory. This is necessary to ensure that the bootloader is started when the system boots. The “control mode” executable is stored at a fixed location after the bootloader. The remaining space can be used to store user apps or data files. Such files are managed through the use of an index table at the end of the flash memory. It stores information about the start and size of files stored in the flash memory. The start of files is always aligned to the size of the flash memory blocks. Binary application files are parsed and loaded into memory. In this way, the bootloader can load and execute user applications.

Since the core EoT device does not have typical I/O capabilities, such as screen and keyboard, and we aim at making it easy-to-use and easy-to-deploy, the perfect companion is a smartphone or tablet. In the typical scenario, users will download apps in their smartphones to interact with EoT devices. Therefore, each EoT-based application will typically have two binaries:

- the vision application stored and executed in the EoT device
- interface/configuration application running in the smartphone/tablet (or a desktop/laptop computer)

While the former is handled by the bootloader, the latter relies on device communication mechanisms and companion applications for other platforms.

#### 4.1.2. SD Card Management

A fully-fledged filesystem is available on the device micro-SD card. The SD card is physically accessible on the EoT board. To remove the card, all it takes is to push the card slightly to eject it. These SD cards can be read in modern laptops, which come with an SD card slot. This makes the presence of an SD card in EoT especially sensitive. In order to ensure security of the card, two additional features have been implemented for extra security: encryption and file shredding. All cards’ contents are stored in an encrypted form. File-level encryption was implemented using AES-CTR with a 128-bit key length. On the other hand, apart from the basic ‘delete file’ function available in the SD card filesystem API, a ‘shred file’ functionality is available in EoT that overwrites the file with zeroes prior to its deletion. Needless to say, this optional operation takes longer than the basic ‘delete file’.

#### 4.1.3. Audio Input and Output

Again, given the limited interaction capabilities of the device (as compared with, say, smartphones), providing audio feedback was considered very useful in some scenarios, and therefore, a module that handles the EoT’s audio chip for playback and recording audio signals is available. Encoding and decoding with the Opus audio format is supported. Thus, audio data can be read from and written to files in the OGG container format. Encoding and decoding of this format is based on the reference implementation of the codec. The OGG container format is processed using the libogg reference implementation. The audio chip is configured by this module through the I2C interface. Audio data are transmitted through the aforementioned interface transparently to the programmer by the use of the module functions.

#### 4.1.4. Power Management

Different components of the EoT device can be selectively activated/deactivated to reduce power consumption. The power of the Myriad SoC is controlled by the so-called power islands. In total, there are 20 power islands. Each power island controls the power supply of a certain component in the chip. The SHAVE drivers will automatically turn off SHAVE islands when not in use. Additionally, the WiFi chip has automatically-managed low-power modes.

#### 4.1.5. Motor Control, Drone Control

In order to communicate with a wide range of devices such as robots and in general with other sensors and actuators, this module encapsulates the hardware details of the communication into an easy-to-use library. The module makes it possible to control small robots and drones; open and closed doors as required; monitor devices such as fridges, vending machines; or control systems like air conditioning and lighting, which might not have access to the Internet or wireless connectivity. The module is divided into two main libraries: a library for controlling ground vehicles (which includes functions such as: ‘move forward’, ‘move backward’, ‘tank-turn left’, ‘tank-turn right’ and ‘stop’) and a library for controlling drones by means of the MAVLink protocol [27]. The first one is a C/C++ library, which was initially developed for Arduino, ported to Raspberry Pi and finally ported to the EoT device, while the second one is a port of the C/C++ MAVLink library that has been modified to send and receive the information through the EoT’s UART communication port.

### 4.2. Communications

#### 4.2.1. WiFi and MQTT

When the EoT device is powered on for the fist time, it creates an ad-hoc WiFi, necessary to allow connection for the configuration of the device even without WiFi infrastructure. This is because the EoT device cannot connect to an existing access point directly since it has no means for specifying the parameters needed for connection. Another device should be used to establish connection with it, allowing one to configure each EoT device one by one. In this mode, an EoT device can be made to connect to an existing access point allowing them to connect to each other (with or without infrastructure) or to connect to the Internet if necessary. Security is handled by an encryption protocol used in the ad-hoc WiFi (WPA2).

Efficient options exist for low-power connectivity [28]. Some examples are ZigBee and the more recent Bluetooth LE (low-energy). The approach proposed for developing the EoT device will focus on using TCP/IP over WiFi, since latest WiFi modules (also the one used in EoT) include low-power features focused on the Internet of Things, having low-power standby modes and short wake-up times.

Low-power operation not only depends on hardware. Software can also play a role. In terms of communication, the protocol used is crucial. EoT communications are based on the TCP/IP stack and the MQTT protocol [29]. Devices that support the MQTT protocol can open a connection and maintain it using very little power while receiving commands with as little as two bytes of overhead. While the well-known HTTPS protocol is slightly more efficient in terms of establishing connection, MQTT is much more efficient during transmission [30]. MQTT implements an extremely efficient and scalable data distribution model; therefore, while HTTP is point-to-point, MQTT allows distributing 1-1 or 1-to-n using the publish/subscribe mechanism. Thanks to these advantages, MQTT is being increasingly used in several mobile apps like Facebook Messenger.

The classic approach followed with MQTT is that of an embedded client connecting to an MQTT server (broker) in the cloud [31]. In this scenario, the server has to be leased by the user, or else it has to be installed on a locally-managed server. EoT’s approach differs in the sense that each EoT device can act as a broker, not having to connect to an external server. This fact allows other EoT devices to subscribe to another EoT. Furthermore, a device used to configure the device can also subscribe to it and receive information through MQTT messages, although it can also ‘publish’ configuration commands (see below) for the device.

#### 4.2.2. Control Mode: “Pulga”

“Pulga” [32], meaning flea in Spanish, is the proposed MQTT broker implementation for EoT devices. Its name derives from Mosquitto [33], which is a well-known Open Source MQTT v3.1 message broker developed by Roger Light. The main idea behind the development of Pulga, as opposed to Mosquitto, is to be a lightweight broker designed to be run in embedded systems.

**Figure 11:**
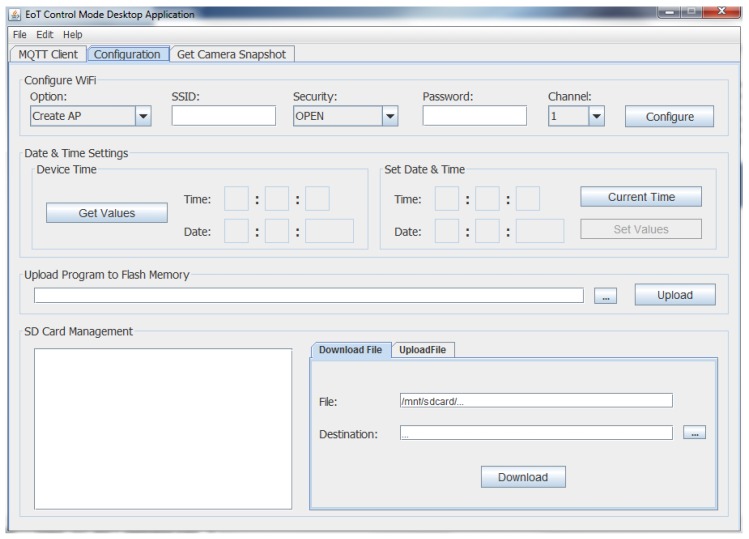
EoT configuration application screenshot (desktop version).

Pulga includes other functionalities that a typical MQTT broker does not have, including the possibility of defining ‘restricted’ topics, allowing new uses for the MQTT protocol. When a Publishmessage is received, Pulga detects if its topic is one of those defined as ‘restricted’ and then acts as the programmer defines. This approach allows the user to have additional functionality, to name a few: send/receive files divided in several binary messages and configuration/control of the EoT device by defining a configuration topic. Following this approach, two applications have been developed for basic device configuration (one for a PC and another one for Android devices) (Figure 11).

The functions provided in the Controlmode are:

- Change from AP mode to Station Modeand connect to another existing AP
- Upload and flash a user application
- Upload data to SD card in EoT
- Download data from SD card in EoT
- Change network password
- Remove network password
- Request a camera snapshot
- Update EoT’s clock/time with the client’s clock/time (clock/time is strictly needed, for example to store timestamps on events)

Apart from being used in Pulga itself, the MQTT communication protocol is also available to user applications.

#### 4.2.3. Video Streaming

**Figure 12:**
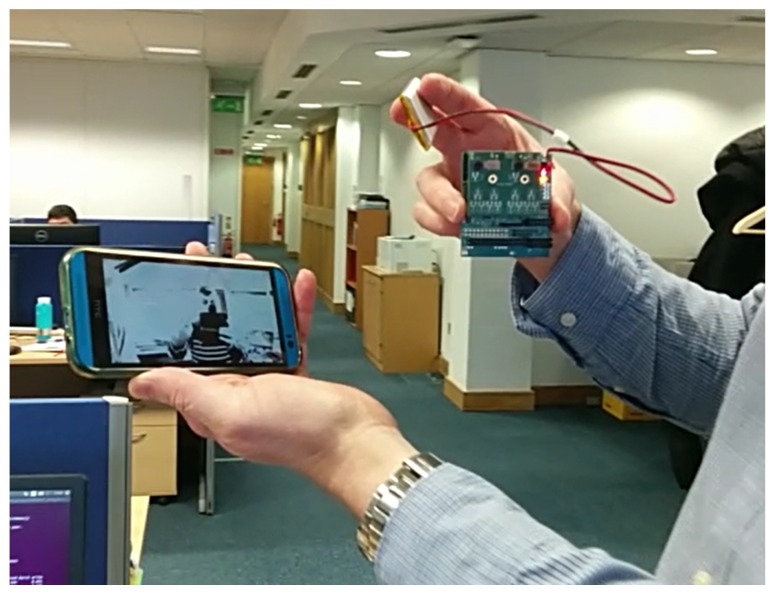
Battery-operated EoT streaming video to an Android smartphone.

The EoT device will also provide a means to stream raw video since in some applications “seeing” is as important as “watching”. In other words, even if automatic image analysis is done, there will be times at which human operators will need to see what is happening and what is being captured by the camera. Streaming allows integration with existing services, such as video surveillance clients and cloud-based video analytics. Streaming may be also useful to configure or monitor the vision application from an external device like a tablet or PC. For all of these reasons, EoT also implements the Real-Time Streaming Protocol (RTSP) typically used for video streaming in IP cameras; see Figure 12.

RTSP is designed to control streaming media servers. The protocol is used for establishing and controlling media sessions between end points. Similar to HTTP, RTSP defines control sequences to facilitate real-time control of the playback of media files such as play and pause. Furthermore, contrary to HTTP, which is stateless, RTSP has a state defined by an identifier, which is used when needed to track concurrent sessions. Moreover, both protocols use TCP to maintain an end-to-end connection, and while most RTSP control messages are sent by the client to the server, some commands travel from server to client.

Streaming with RTSP/RTP will be controlled by the application running in the EoT device and/or externally via MQTT commands sent to it. This allows using the energy-consuming streaming only for specific applications and only when it is needed. The main video streaming parameters (resolution, frame rate and quality) are also under programmer control. Note that in the “see” regime, the processor will be executing mostly codec software, while in the “watch” regime, it is used for computer vision.

Since the EoT RTSP only needs to support streaming and its status control, the developed service implements only the most important features needed to work in order to save memory, power and be more efficient. The RTSP server controls connections and streaming using TCP sockets for the communication with clients. It allows multi-client streaming. In this case, the maximum number of clients is limited by the maximum number of different sockets that the WiFi chip can support. The different options implemented once the connection between the server and the client is established are: OPTIONS, DESCRIBE, PLAY, SETUP and TEARDOWN.

#### 4.2.4. Push Notifications

**Figure 13:**
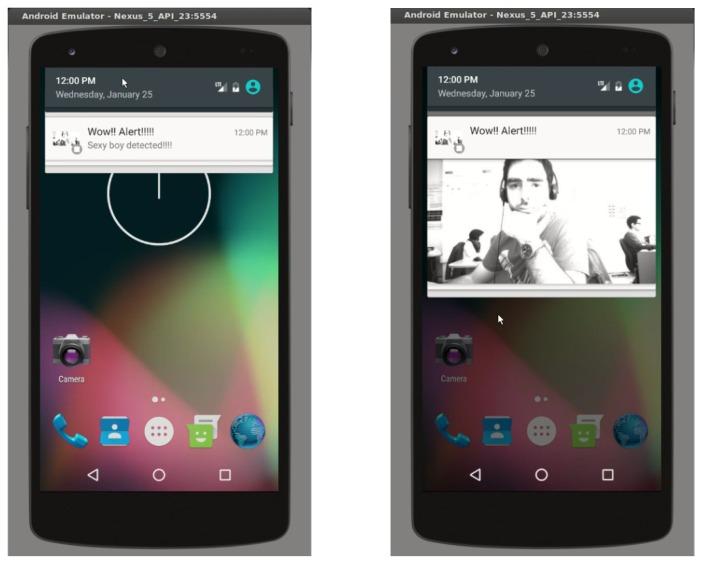
Push notification received on an Android smartphone. Left: prior to the user opening it; right: after touching on the notification.

The EoT device can send push notifications to a user’s smartphone. This is especially useful for applications in which some sort of alarm must be sent to a user. In order to achieve this functionality, EoT uses the Google Firebase platform to generate push notifications. Notifications can include an image; see an example in Figure 13.

### 4.3. Computer Vision

EoT provides the programmer with a wide array of vision libraries that implement common methods used in CV, such as: colour histogram matching, keypoint matching, sparse optical flow, rotation-invariant face detection and CNN inference. In addition to all of the aforementioned functionalities, well-known libraries, such as OpenCV [34], libccv [35] and Quirc [36] are part of the EoT framework. For high performance and low-power computer vision, a library called MvCv is also available, which takes advantage of the various features of the Myriad architecture to achieve maximum performance with minimum power.

On the other hand, while most of the processing in EoT will be local, EoT also provides a means to offload processing to the cloud. In this respect, a library providing native access to the powerful Google Cloud Vision API [37] has been developed. Use of the API is done via HTTP and SSL. This service should be useful for more demanding analysis operations, which cannot be done in the embedded device (or for which the cloud version provides the best option). The API encapsulates machine learning models that can learn and predict the content of an image. At the time of writing, the API includes:

- Detect categories of objects (flowers, animals, etc.); the API improves over time as accuracy is improved and new categories are introduced
- Detect inappropriate content from adult to violent content
- Find celebrities, logos or news events
- OCR, with automatic language identification

#### 4.3.1. OpenCV

OpenCV is the de facto standard CV library, including hundreds of useful functions. OpenCV 1.0 has been ported to the EoT device. Additionally, functionality has been added so that later (much larger and complex) OpenCV versions can be executed in an external server. For this, a free Pythonanywhere https://www.pythonanywhere.com/ server has been used. This is a free service, which provides a server with web2py and OpenCV 2.4.3 in which it is possible to program any computer vision capabilities. The EoT device sends an image to the server, which processes it (using any OpenCV version) and returns metadata. It is also possible to create your own processing server using, for example Python, Django and any OpenCV version, application or library.

#### 4.3.2. Libccv

Libccv is a minimalist open-source computer vision library for embedded devices. Libccv includes several modern computer vision algorithms, such as:

- Image classifier
- Frontal face detector, object detectors for pedestrians and cars
- Text detection algorithm
- Object tracking algorithm
- Feature point extraction algorithm

**Figure 14:**
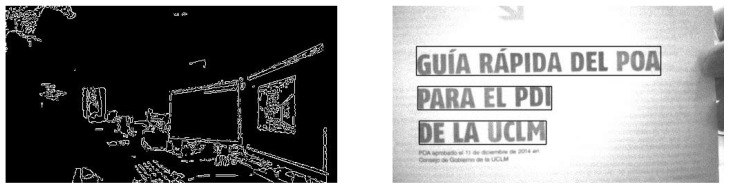
Libccv examples running in EoT. Left: Canny edge detection. Right: text detection in images.

Figure 14 shows two examples that used Libccv. The library has been ported to the EoT device with some limitations (JPEG not supported, reading/writing of sqlite3 files not supported).

#### 4.3.3. Quirc

**Figure 15:**
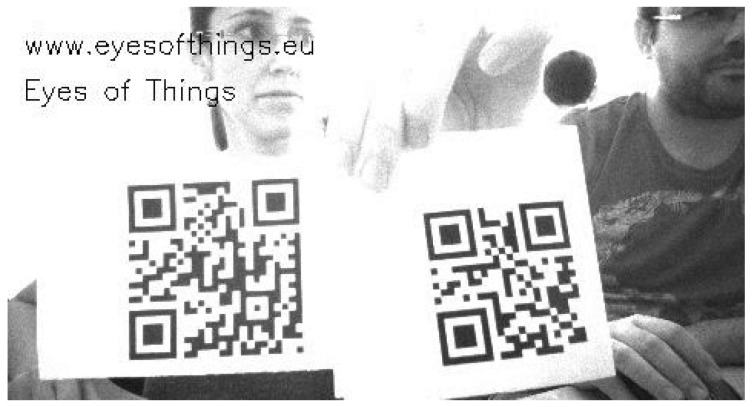
QR code recognition. In this image, two QR codes are recognized.

Quirc is a library for extracting and decoding QR codes in images. It is fast enough to be used with real-time video (extracting and decoding from VGA frame takes about 50 ms on a modern ×86 core). Other features that make it a good choice for the purpose of EoT are: it has a very small memory footprint (one byte per image pixel, plus a few kB per decoder object); it is BSD-licensed with almost no restrictions regarding use and/or modification; and it is easy to use, with a simple API described in a single commented header file. Figure 15 shows an example of Quirc running in EoT.

Even though this library was not optimized, performance is very satisfactory using only the LeonOS processor of the Myriad 2; see Table 1.

#### 4.3.4. MvCv

The MvCv library is part of the Myriad’s MDK. It has been optimized to take full advantage of the processor capabilities. One of the key optimisations is to operate on stripes or tiles from a frame rather than going round trip to DRAM each time, which greatly reduces power and maximizes performance. The MvCv library has been used by Movidius across dozens of projects and contains hundreds of commonly-used CV functions, including: a high performance multi-frame point tracker called vTrack, visual odometry, HoG, Kalman/ROI, H.264 video-codec, SGBM, BLIS, JPEG codec, image warp, RANSAC, FREAK and various linear algebra solvers.

#### 4.3.5. Colour Histogram Matching

Colour histogram matching is a simple method for comparing the visual similarity of images or image regions [38]. Despite its simplicity, it can be quite effective, especially if an appropriate colour space is chosen. The process consists of two parts. First, the colour histograms of the images or image regions, which shall be compared, are computed. Then, the matching score is computed based on a histogram distance metric. The histogram matching module implements the computation of the colour histograms, as well as different distance metrics to compute the distance score. Currently, the histogram intersection, the Hellinger distance and the earth mover’s distance are implemented.

#### 4.3.6. Keypoint Matching

The ability to detect and match keypoints in two different images is a fundamental functionality in many computer vision algorithms. Algorithms involving keypoint matching generally distinguish three different steps: feature detection, feature description and feature matching [39]. In feature detection, the input image is searched for particular points with the following properties: the same 3D point should be detected under various viewpoints, scale changes and lighting conditions (repeatability), and the detected point should have a unique visual signature (description). For this reason, keypoint detectors usually detect points in an image with sufficient texture and corresponding to positions of high frequency, usually referred to as corners. A feature descriptor is an algorithm that computes the signature of a specific point in the image based on its visual appearance. The desirable properties of a descriptor are the uniqueness of the description and invariance of the description under various changes (scale, brightness, rotation, etc.). The descriptor itself is usually represented as a fixed-size vector of real numbers, but recent descriptors rather produce binary codes to increase the efficiency of the matching process. Feature matching amounts to the computation of a distance between feature descriptors. The distance must be chosen such that the difference between descriptors of the same 3D point seen under various conditions is generally small, whereas the difference between descriptors of different 3D points should generally be higher. In many cases, the L1 or L2 distance can be chosen, but more complex distance definitions are possible.

In the last few decades, many different algorithms for keypoint detection, description and matching have been proposed with various properties [40]. Recent developments tend to favour fast detectors based on simple pixel intensity checks, as well as binary descriptors, as they are more suited to a large number of keypoints while keeping the matching process tractable. Among the recently-published algorithms, EoT supports the BRISK keypoint algorithm, as it proved to be the most promising in terms of matching capabilities. BRISK uses a scale-space version of AGAST3 as the keypoint detector, a binary code based on the difference of pixel pairs as the descriptor and a Hamming distance for matching.

#### 4.3.7. Rotation-Invariant Face Detector

**Figure 16:**
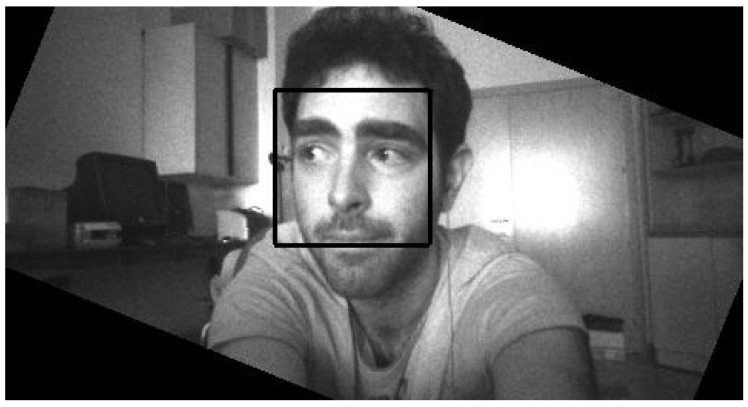
Rotation-invariant face detection. In this example the user (pictured in the image) is holding the EoT and tilting it.

EoT is a mobile device and as such it can be worn or otherwise rotated in space. In order to detect faces even in non-canonical orientations, a rotation-invariant face detector was implemented. The algorithm is based on obtaining the device’s angle in space, rotating the input image and then applying an upright face detector. Figure 16 shows an example of a face detected by the algorithm and the rotation of the frame (hence the black border).

#### 4.3.8. Sparse Optical Flow

Optical flow is the pattern of apparent motion of image objects between two consecutive frames caused by the movement of object or camera [41]. It is a 2D vector field where each vector is a displacement vector showing the movement of points from the first frame to the second. Although it is possible to obtain this functionality by means of the OpenCV library, an optimized implementation called vTrack is also provided. Both implement Lucas–Kanade point tracking, although the OpenCV solution only makes use of the Myriad 2 LeonOS processor, while vTrack uses also the SHAVEs, having better performance.

To minimize the runtime and improve the tracking quality, the vTrack algorithm has a configurable number of SHAVEs. The user can minimize the latency of the data by using more SHAVEs. Moreover, using gyro data, the algorithm is capable of estimating the new position of the features. With gyro assist, the tracking quality and speed are improved. In addition, the algorithm can process multiple images in parallel.

Note that, in terms of optimized performance, from the beginning of the project, the goal was two-fold. On the one hand, EoT was to provide optimized functionality for vision tasks (even though this may be restricted to a few low-level primitives). On the other hand, we explicitly aimed at improving productivity and fast development (meaning direct access to useful and easy-to-use rich functionality that is not optimized). While the proprietary MvCV library is optimized, the library only provides low-level functionality and primitives for the developer. That is the maximum level of optimization present in EoT, which has come at the expense of huge optimization efforts made by the SoC designer, and is restricted to a few dozen low-level routines. Other software developed in EoT has been optimized too using the SHAVE processors, such as the face detection routines and the tiny-dnn CNN engine. Finally, other large libraries such as OpenCV are not optimized. In summary, our objective was to provide the developer with different points in the optimization/functionality trade-off.

### 4.4. Deep Learning

Many large companies have invested heavily in machine intelligence for vision and other applications over the past five years [42]. To date, these applications have largely been confined to the cloud. Given the difficulties in scaling vision-based solutions, which include power dissipation, latency, continuity of service, bandwidth and privacy, there is huge interest in migrating such applications in whole or in part to the network edge. The deep learning paradigm is powering most of those applications.

The driving force behind this move towards deep networks has been the huge gains in accuracy that have been made due to the introduction of CNNs on benchmarks like ImageNet compared to the previous incumbent methods. The broad deployment of CNNs has tracked the massive computational power afforded by the introduction of Graphics Processing Units (GPUs).

As an evolutionary step in neural networks, they are becoming a key method in applications, such as vision processing, handwriting recognition, robotics and automotive self-driving systems. Conceptually, CNNs are similar to ordinary neural networks in the sense that they are composed of a multitude of neurons with trainable weights and biases. Neurons individually receive some inputs and perform dot products (optionally followed by non-linearity). However, the whole network still introduces a single differentiable score function from the raw input image pixels to the class scores.

#### 4.4.1. Fathom Framework

**Figure 17:**
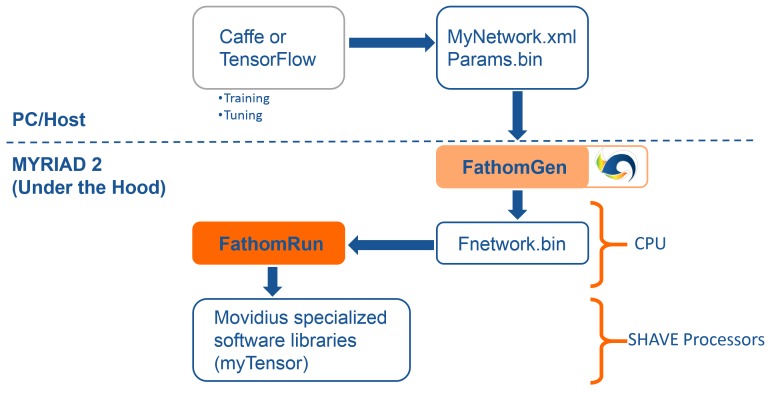
Fathom deep learning framework.

In order to address the deep learning paradigm, a framework called Fathom is available in EoT that implements CNNs of various configurations at ultra-low power targeting the Myriad SoC. The Fathom framework accepts representations of trained networks from Caffe or TensorFlow, as shown in Figure 17. Fathom parses these and optimally translates them to run on the Myriad 2 architecture. For complex networks such as GoogLeNet, Fathom enables performance at a nominal 15 inferences/s with fp16 precision.

The idea behind the Movidius Fathom™ framework is to take an existing trained network from a CNN framework like Caffe or TensorFlow and translate and adapt it for use with a Myriad-based device. In this way, the current design flows used by deep learning data scientists to develop networks are unchanged, and they can continue to use the flows, datasets and associated scripts they have used historically without modification to quickly and easily port the networks trained in the cloud or GPUs to the EoT platform.

Fathom sits on top of the MvTensor library, which in turn is built on top of the MvMatMul library. The MvTensor library provides highly efficient 3D/4D convolution with an initial focus on strided small convolutions required by GoogLeNet. The library uses direct methods and matrix multiplication (MvMatMul) and supports both fp32, fp16 and 8-bit operations sustaining up to 80 GFLOPs at half precision.

#### 4.4.2. Tiny-Dnn

While the Fathom framework is proprietary by Movidius, EoT also implements tiny-dnn. tiny-dnn is a C++ implementation of deep learning suitable for use on limited computational resources, embedded systems and IoT devices [43]. The library is easy to integrate with other applications and can import pre-trained Caffe models. This library has a permissive BSD 3-clause license. The library has been included in recent versions of the well-known OpenCV library.

### 4.5. Scripting

**Figure 18:**
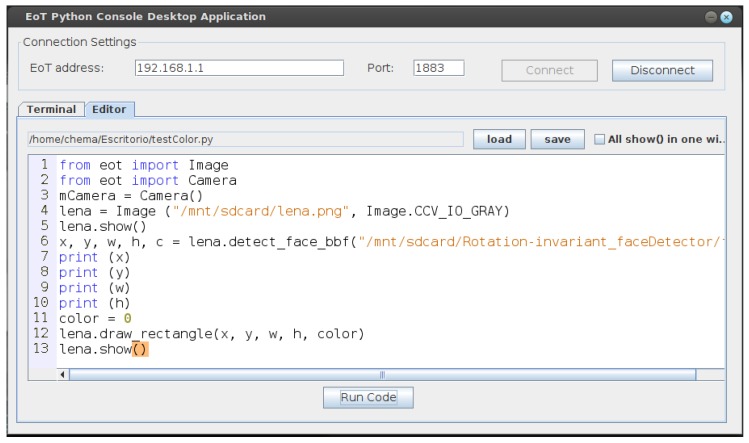
EoT MicroPython remote terminal and editor showing a simple vision application.

All development for EoT uses C/C++. Even though some level of code optimization is often necessary to achieve a good performance, in many cases, no optimization is needed. Flexibility and ease of use are thus desirable. The Python programming language’s flexibility and ease-of-use have made it wildly popular in academia and in general in many realms where speed of development and flexibility are more important than performance. Python is an interpreted language, which means that it is not in principle a good fit for embedded systems. In the past, a number of attempts have been made at porting it to embedded platforms. MicroPython is currently the most successful port [44]. The EoT device supports MicroPython. A REPL system is already available on top of the MQTT communication protocol. This allows sending of instructions to and receiving responses from the EoT remotely, from a PC or tablet; see Figure 18.

## 5. Results

The Bill of Materials (BoM) for the final Form-Factor Board (FFBoard) has a total of 367 components. The total cost of the BoM components including camera is €115. We sourced and ordered components for 30 prototype boards from a number of different vendors (RICOH, Movidius, SAMTEC, Würth, Digi-Key, Mouser, etc.). This cost may be reduced by a more careful selection of sources and by considering volume amounts.

**Figure 19:**
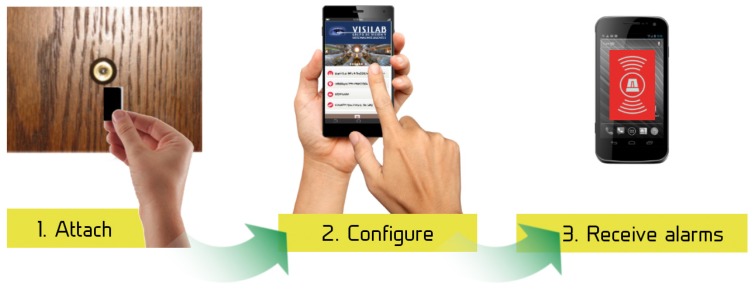
Peephole demonstrator.

**Figure 20:**
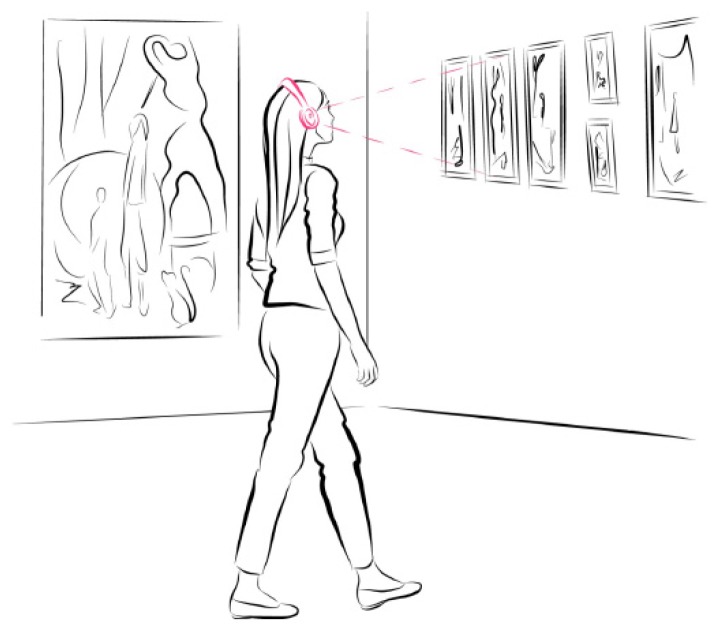
Museum audio tour demonstrator.

A number of demonstrator applications are being developed with the platform:

- Peephole door viewer, Figure 19: Before leaving the home, the user will attach the EoT device to the peephole. The device will continuously monitor for the presence and/or suspicious activity at the door (or simply someone knocking at the door), sending alarms and pictures via Internet (assuming home WiFi is available). Tampering detection (i.e., an attempt to cover the peephole) will be also implemented and will also generate an alarm.
- Museum audio tour, Figure 20: The EoT device is worn (headset) by a visitor, and it automatically recognizes paintings in a museum, providing additional audio information, all without the user’s intervention.
- Versatile mobile camera: The device will capture and save images when an interesting event occurs. Images saved can be retrieved and post-processed using cloud support.
- Smart doll with emotion recognition: An EoT device is embedded inside a doll’s head (or torso). Facial emotion recognition runs locally in the device so that the doll can assess the child’s emotional display and react accordingly (with audio feedback).

In the following, we provide more details about the last two demonstrators.

### 5.1. Versatile Mobile Camera

**Figure 21:**
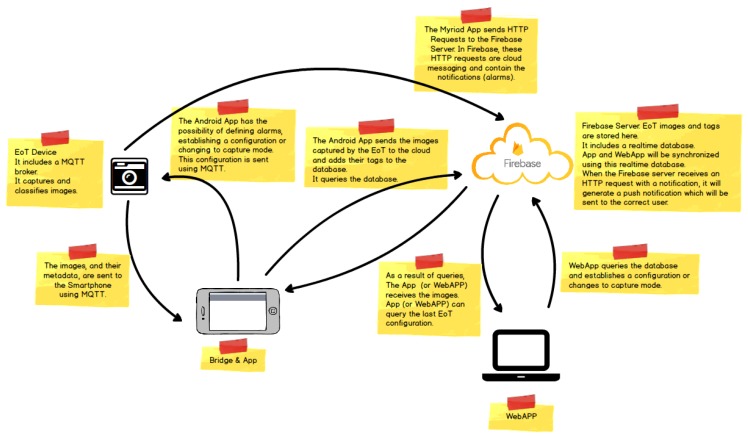
Architecture of the versatile mobile camera demonstrator.

Figure 21 shows the architecture of the demonstrator. On the left, the EoT device can work in one of two modes. In the ‘normal’ mode, the camera captures a snapshot at regular intervals (configurable by the user). Snapshots are stored on the SD card. In the ‘smart’ mode, the camera performs an analysis of the image, trying to detect ‘key events’ in which the user is interested. Whenever one or more of the key events is detected, the image is stored in the SD card, along with text information (tags) indicating the events recognized.

The whole demonstrator leverages the Google Firebase cloud platform. Firebase is a cloud infrastructure and tools to help developers build apps. The demonstrator Android app allows on to retrieve images from the EoT SD card, along with their associated tags. This app also allows one to change the EoT device configuration, setting key events, alarms, etc. When the user retrieves images and associated metadata, they are automatically uploaded to a Firebase database. The Android app has an embedded WebView. A WebView is a component that allows a native app to show and run a web application. In other words, it is a browser bundled inside of a mobile application producing what is called a hybrid app. Using a WebView allows mobile apps to be built using web technologies (HTML, JavaScript, CSS, etc.), but still package it as a native app. This runs a webapp for managing the images in the Firebase database, providing additional functionality. This webapp can be also used from any standard web browser. The webapp provides a GUI displaying images in different modes and providing search functionality. This webapp also provides a series of API-based activities, presented in a Zapier/IFTTT manner to allow further processing or distribution of images. Some examples of this could include:

- IF Angry face from EoT-1 email to my_address@my_company.com
- IF Happy face from EoT-2 post to Facebook
- IF Large Face from EoT-3 send to Google Vision API, add results to Firebase and Email results to my_address@my_company.com
- Create/Share my Diary
- Create a collage by: Face Type, Date Range, ...
- Create a montage by ...
- Send to Zapier

**Figure 22:**
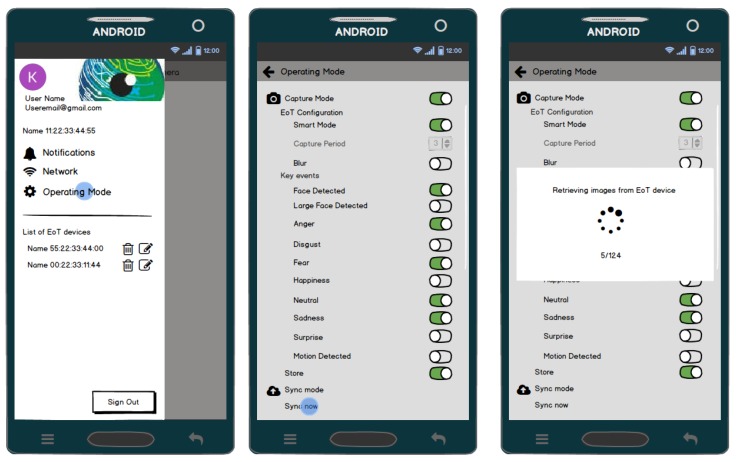
Sample screens of the demonstrator’s Android app.

Firebase also provides user authentication, as well as push notifications (used in alarms) to the smartphone app. Figure 22 shows some screenshots of the Android app.

**Figure 23:**
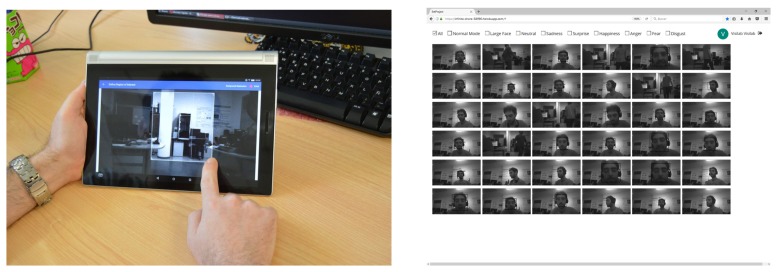
Left: defining a region of interest for motion detection. The region configured is sent to the EoT device through MQTT over WiFi communication. Right: access to captured images from a PC web browser. The images were previously retrieved from the EoT device and uploaded to the cloud database.

Figure 23 shows the ROI definition running in a tablet and interaction with images from a PC web browser.

### 5.2. Smart Doll with Emotion Recognition

**Figure 24:**
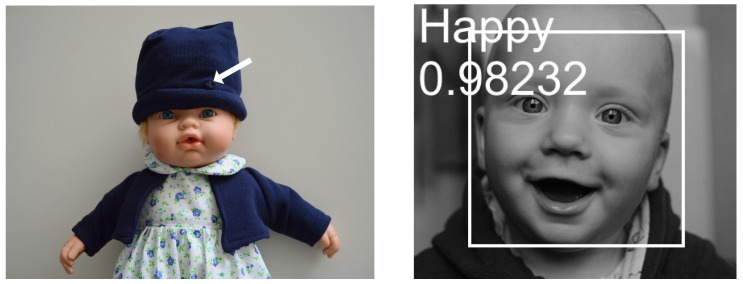
Smart doll with emotion recognition. Left: The camera was in the head, connected through a flex cable to the board and battery inside the body. Right: emotion recognition.

**Figure 25:**
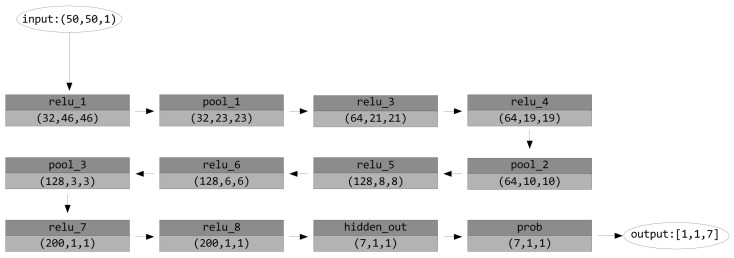
Emotion recognition network.

This demonstrator (see Figure 24) leverages the rotation-invariant face detector, as well as the CNN inference engine. Invariance to rotation is needed in this case since the child may be holding the doll. The face detector crops and resizes the face, which is then fed to the CNN classifier. A 12-layer trained network generated by the company nVISO is used to do the emotion recognition; see Figure 25. The trained network occupies 0.9 MB of memory.

**Figure 26:**
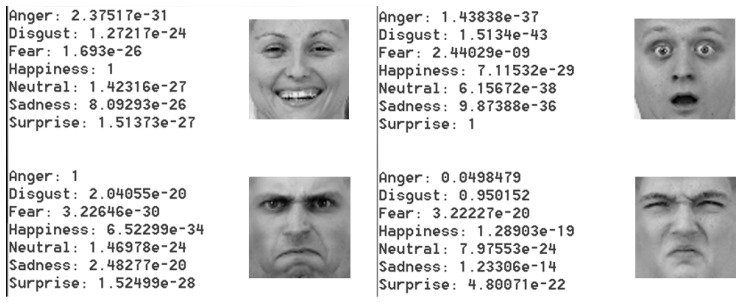
Four sample emotion images and the output given by the network.

Figure 26 shows some example outputs given by the network.

Table 2 shows the times taken by the inference part of the demonstrator, in three different cases. Loading the network weights from the device SD card takes around 1 min (this has to be done at the start only).

The recognized emotions are optionally stored on the SD card and can be later retrieved from a smartphone or tablet. The audio feedback is based on canned sentences and sounds.

This demonstrator requires battery-operated mode, and thus, power consumption is fundamental. Table 3 shows qualitative measures of power consumption.

**Figure 27:**
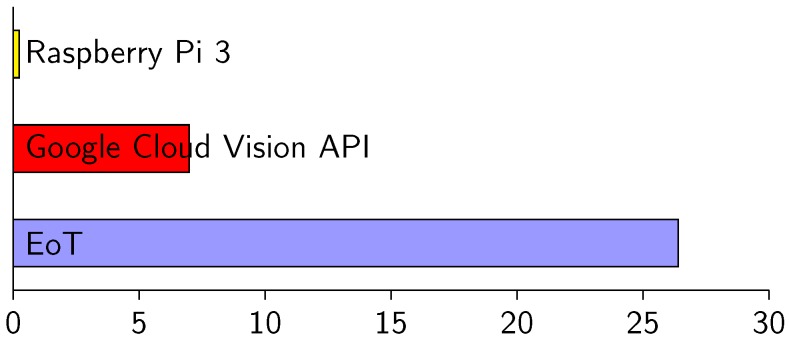
Efficiency for the task of face emotion recognition, in images/s/Watt.

Figure 27 shows efficiency values in three scenarios. Efficiency is measured here in terms of images per second per Watt. In the three cases, the task was facial emotion recognition. In one of the cases, we used the Google Cloud Vision API for the task. Using this cloud processing service, the emotion label was returned with an average latency of 89 milliseconds (100 Mbps WiFi infrastructure). Even with the low-power WiFi chip used, this consumes 1.61 W. The efficiency in this case is 6.98 images/s/Watt, whereas the efficiency of local in-device processing for the same task was 26.4 images/s/Watt (last row of Table 3). Facial emotion recognition is a relatively complex task. If we consider simpler tasks (face detection, optical flow, edge detection, etc.), the difference in efficiency of local vs. cloud processing is even higher because time spent during communication becomes the most important factor. On the other hand, it is obvious that it will be always possible to find sufficiently complex tasks that can be executed more efficiently in the cloud. In fact, one of the goals in EoT was to facilitate interaction with cloud-based vision processing, with the assumption that for some applications, this will be the best option.

Overall, the results presented show a positive outcome in terms of cost, size, power consumption, programmability and interaction with mobile devices and cloud services. The possibility of having state-of-the-art machine learning inference for 1.1 W, running locally, has been also demonstrated.

## 6. Conclusions

In the context of the Internet of Things and the growing importance of cognitive applications, this paper has described a novel sensor platform that leverages the phenomenal advances currently happening in computer vision. The building elements have been all optimized for efficiency, size and flexibility resulting in the final EoT form factor board plus a stack of useful software. Apart from hardware and architectural elements, software and protocols used have been also optimized. The platform software and hardware specs are open and available at the project website. There are options for board evaluation and for support. Both hardware and software repositories include wikis with documentation and examples. The information and support provided allow creating a custom design if needed.

EoT is an example of an IoT sensor exemplifying a number of tenets:

- As sensor capabilities improve, intelligence must be brought to the edge
- Edge processing alone is not sufficient; a concerted effort with cloud elements will still be needed in many applications
- In order to reach the potential audience and usage expected in IoT, complex (if powerful) embedded systems require flexibility, added-value and close interaction with other widely-used products such as smartphones/tablets
- Low-power operation requires not only low-power hardware, but also careful software design and protocol selection

The results so far suggest that the platform can be leveraged by both industry and academia to develop innovative vision-based products and services in shorter times. The demonstrators described above are just an example of the platform’s capabilities, and the platform is expected to be particularly useful for mobile robots, drones, surveillance, headsets and toys. In future work, we expect that more sample applications will be developed by both consortium and external partners, particularly combining many EoTs in distributed applications.

## Figures and Tables

**Table 1:** Times for QR code detection and recognition.

Image Dimensions (w × h)	No. of Codes	Time
500 × 500	1	1341 μs
400 × 300	2	3502 μs
50 × 50	1	1254 μs

**Table 2:** Times taken by the inference part of the demonstrator. The EoT DevBoard is the second iteration board (i.e., Figure 1, board in the middle). The Fathom case used only four of the 12 SHAVEs available.

Hardware	Software and precision	Time (ms)
High-end laptop	tiny-dnn, 32-bit FP	190
EoT DevBoard	tiny-dnn, 32-bit FP	700
Movidius devel. board	Fathom, 16-bit FP	17

**Table 3:** Qualitative power consumption measures. All radio communication components were deactivated in the cases shown, except for the EoT Form-Factor Board (FFBoard). Both EoT DevBoard and Movidius devel. board used the IMX208 camera (see Figure 8). The Fathom case used only 4 of the 12 SHAVEs available and half precision. Smartphone model: Xiaomi Mi5.

Platform	Task	Average Power Consumption (W)	Batt. Life (h) (3.7 V, 4000 mAh)
Android smartphone	idle	3.35	
	face detection	5.82	2.5
Raspberry Pi model 3	idle, no cam	1.34	
	face detection	1.94	
	face emotion recognition	2.06	7.1
EoT 1st Prototype	idle, no cam	1.32	
EoT DevBoard	idle, no cam	1.28	
	face detection	2.24	
	face emotion rec. w. tiny-dnn	2.22	
EoT FFBoard	idle	0.30	49
	face emotion rec. w. tiny-dnn	1.10	13.4
Movidius dev. board	face emotion rec. w. Fathom	2.22	

## Abbreviations

The following abbreviations are used in this manuscript:

AES	Advanced Encryption Standard
AGAST	Accelerated Segment Test
AP	Application Processor
BLIS	BLAS-like Library Instantiation Software
BRISK	Binary Robust Invariant Scalable keypoint
CNN	Convolutional Neural Network
FPS	Frames Per Second
FREAK	Fast Retina Keypoint
GPIO	General-Purpose Input-Output
GFLOPS	Giga (i.e., billion) Floating Point Operations Per Second
HOG	Histogram of Oriented Gradients
IFTTT	IF This, Then That
IMU	Inertial Measurement Unit
ISP	Image Signal Processing
JSON	JavaScript Object Notation
LVDS	Low-Voltage Differential Signalling
MQTT	Message Queue Telemetry Transport
OCR	Optical Character Recognition
OEM	Original Equipment Manufacturer
PCB	Printed Circuit Board
PMIC	Power Management Integrated Circuit
PRNU	Photo Response Non-Uniformity
PWM	Pulse Width Modulation
RANSAC	Random Sample Consensus
REPL	Read-Eval-Print Loop
RISC	Reduced Instruction Set Computer
RTEMS	Real-Time Executive for Multiprocessor System
RTOS	Real-Time Operating System
RTSP	Real-Time Streaming Protocol
SBC	Single Board Computer
SGBM	Semi-Global Block-Matching
SoC	System-on-Chip
SSL	Secure Socket Layer
VLIW	Very Long Instruction Word
VPU	Visual Processing Unit
